# Establishment of Nude Mice Lacking NK Cells and Their Application for Human Tumor Xenografts

**DOI:** 10.31557/APJCP.2021.22.4.1069

**Published:** 2021-04

**Authors:** Jutatip Panaampon, Kenichi Sasamoto, Ryusho Kariya, Seiji Okada

**Affiliations:** *Division of Hematopoiesis, Joint Research Center for Human Retrovirus Infection and Graduate School of Medical Sciences, Kumamoto University, Kumamoto, Japan. *

**Keywords:** Nude mice, Jak3 deficient mice, natural killer cells, xenotransplantation, immunocompromised mice

## Abstract

**Objective:**

Nude mice are used as a recipient for human tumor cell xenografts. However, the success rate of xenotransplantation is unsatisfactory due to high natural killer (NK) activity. To overcome this limitation, we established nude mice with no NK cells, and compared the transplantation efficiency with that in nude mice.

**Methods:**

BALB/c Nude Jak3-/- (Nude-J) mice were established by crossing BALB/c Nude mice and BALB/c Jak-3-/- mice. Hematopoietic malignant cell lines (BCBL-1 and Z138) were implanted subcutaneously to compare the tumorigenicity in Nude-J mice with Nude Rag-2/Jak3 double deficient (Nude RJ) mice and nude mice.

**Results:**

Nude-J mice showed complete loss of NK and T lymphocytes, whereas B lymphocytes remained. Both BCBL-1 and Z138 human lymphoid malignant cell lines formed almost the same sizes of subcutaneous tumors in Nude-J and Nude RJ mice, whereas they formed no or only small tumors in nude mice. Splenocytes from Nude-J mice showed no cytotoxic activity *in vitro*.

**Conclusion:**

Nude-J mice can be a valuable tool for human tumor cell transplantation studies.

## Introduction

Nude mice were the first immunocompromised mouse strain to be discovered, in 1962, and have been used for various cancer studies (Flanagan, 1966). They show abnormal hair growth and defective development of the thymic epithelium caused by spontaneous deletion in the Foxn1 gene (Nehls et al., 1994). Thus, homozygous nude mice lack mature T cells through the thymus, T-cell-dependent B-cell response, and coat hair. Since then, nude mice have been used as recipients for human tumor xenotransplantation (Okada et al., 2018). However, the intact (or rather activated) innate immunity in nude mice, especially NK cells, limits the options for human cancer cell transplantation (Bellet et al., 1979). Therefore, nude mice with additional immunodeficiency have been established to improve the take rate of human tumors in nude mice. Lasat mice (asplenic athymic mice), NIH type 2 nude mice, CBA/N nude mice with X-linked partial B cell deficiencies, nude-beige mice and NOD/Scid nude mice have been established. However, since NK activity is lower but still present in these mice, the take rate of human tumors has not obviously improved (Okada et al., 2018; Okada et al., 2019). 

Jak3, a non-receptor type tyrosine kinase, mediates cytokine receptor signaling through an association with the common ɤ chain of the cytokine receptors, and is crucial for lymphocyte and NK cell development and function (Vainchenker et al., 2008). Jak3 deficient mice show drastically decreased T and B lymphocytes and complete loss of NK cells, which is same phenotype as common ɤ chain deficient mice (Park et al., 1995; Suzuki et al., 2000). On the basis of these findings, Jak3 or common ɤ chain deficient mice were crossed with lymphocyte deficient NOD/Scid or Rag-2 deficient mice and used as efficient recipients of human cell xenografts (Shultz et al., 2014; Okada et al., 2019). 

In this study, we established Jak3 deficient nude mice (Nude-J mice), and found that Nude-J mice can be the ideal recipients for human hematological tumor cell xenografts. 

## Materials and Methods

*Mice*

BALB/c Jak3-deficient (Jak3-/-) mice were established by crossing C57/BL6 strain Jak3-/- mice (Park et al., 1995) (Center for Animal Resources and Development, Kumamoto University, Japan) with the BALB/c strain for 10 generations. BALB/c Nude Jak3 deficient (Jak3-/-) mice were established by crossing BALB/c Jak3-/- mice and BALB/c Nude mice (purchased from Japan Clea, Tokyo, Japan). BALB/c Nude Rag-2-/-Jak3-/- (Nude RJ) mice were then established by crossing BALB/c Rag-2-/-Jak3-/- mice (Ono et al., 2011) and BALB/c Nude mice (Kariya et al., 2014), and were housed and monitored in our animal research facility according to institutional guidelines. All experimental procedures and protocols were approved by the Institutional Animal Care and Use Committee of Kumamoto University. 

*Histological analysis*

Spleens were fixed with 10% neutral-buffered formalin immediately after removal, embedded in paraffin, cut into 4 μm sections, and stained with Hmatoxylin and Eosin. 

*Cell lines*

The natural killer sensitive mouse lymphoma cell line Yac-1 (obtained from RIKEN cell Bank, Tsukuba, Japan), the human primary effusion lymphoma cell line BCBL-1 (obtained through the AIDS Research and Reference Reagent Program, Division of AIDS, NIAID, NIH), and human mantle cell lymphoma cell line Z138 (obtained from JCRB cell bank, Osaka, Japan), were maintained in RPMI1640 medium (Wako Pure Chemical, Osaka, Japan) supplemented with 10% (v/v) heat-inactivated Fetal bovine serum (Nichirei Biosciences, Tokyo, Japan), 100 U/ml penicillin and 100 μg/ml streptomycin in a humidified incubator at 37°C and 5% CO_2_.

*Flow cytometry*

Mouse splenocytes were stained with anti-mouse (m)CD49b(DX5)-APC (pan NK marker) and mCD122 (IL-2Rβ)-PE, or mCD19-FITC and mCD3-PE (Biolegend, San Diego, CA, USA), and analyzed using FACSCelesta (BD Biosciences, San Diego, CA, USA) to detect murine lymphocytes. Data were analyzed with FlowJo (Tree Star, San Carlos, CA, USA).

*Xenograft mouse model*

BALB/c Nude mice (Nude), BALB/c Nude Jak3-/- mice (Nude-J) or BALB/c Nude Rag-2-/-Jak3-/- mice (Nude RJ) (8–10 weeks old) were subcutaneously inoculated with 1 × 107 BCBL-1 cells or Z138 cells in both flanks. Tumor size was measured using a digital spring-loaded micrometer (Mitsutoyo Co., Kawasaki, Japan). When the tumor size increased, the xenotransplanted mice were sacrificed, and the tumors were removed and weighed. 

*Cytotoxicity assay*

The cytotoxic activity of murine NK cells was measured by flow cytometry. Briefly, target Yac-1, BCBL-1 or Z138 cells were stained with 5 µM of fluorescence labeling reagent, 5-(6)-carboxy-fluorescein succinimidyl ester (CFSE, Dojindo, Kumamoto, Japan), in accordance with the manufacturer’s instructions. 5 x 10^4^ CFSE-labeling target cells were co-cultured with mouse splenocytes (effector cells) at an E:T ratio of 25:1, 100:1, or absence of effector cells in an incubator for 4 h at 37°C in 5% CO_2_. The murine splenocytes were stimulated with 20 ng/ml of recombinant mouse IL-2 (Biolegend, San Diego, CA) for three days before performing the assay. The cells were then stained with Ghost Dye 780 (Invitrogen, Carlsbad, CA) to identify dead cells. The percentage of dead target cells (CFSE+ Ghost Dye 780+) was determined by a FACSCelesta flow cytometer (BD Biosciences). The data were analyzed by FlowJo software. Cytotoxic activity was calculated as follows: A/(A + B) × 100- C (%), where A is the percentage of CFSE+ Ghost Dye 780+ cells, B is the percentage of CFSE+ Ghost Dye 780- cells at each E/T ratio, and C is the percentage of spontaneous Ghost Dye 780+ cells without effector cells (A/(A + B) × 100 (%) at E/T ratio = 0) (Okada et al., 2008).

*Statistical analysis*

The significance of differences observed between experimental groups was determined using Student’s t-test. P values less than 0.05 were considered significant.

## Results

*Characterization of Nude-J mice*

**Figure 1:**
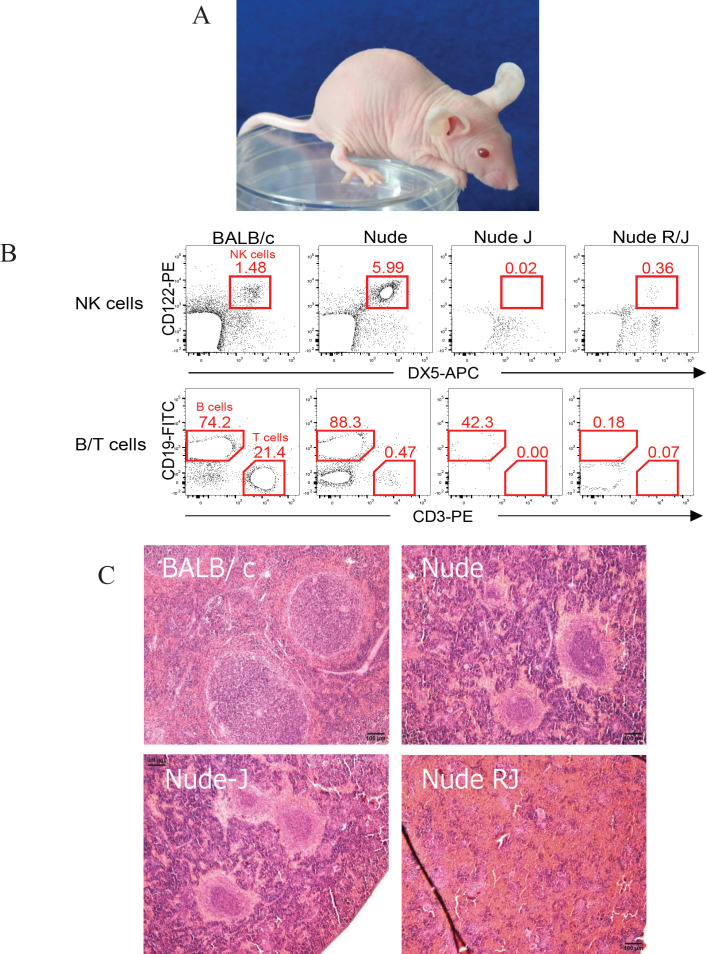
Lack of Coat Hair, Mature T Lymphocytes and NK Cells in Nude-J Mice. (A) Appearance of Nude-J mice. (B) Lack of T lymphocytes and NK cells in Nude-J mice. Spleen cells were stained with anti-mouse(m)CD122 (IL-2Rβ)-PE and mDX5(CD49b)-APC (pan NK marker), or mCD19(B cell marker)-FITC and mCD3(T cell marker)-PE, and analyzed by flow cytometry. No T lymphocytes or NK cells were observed in the spleens of Nude-J mice. (C) Hematoxylin & Eosin staining of the spleen. Nude-J mice and Nude mice keep follicular structure in spleen in contrast to marked reduction of follicles in Nude RJ mice

The generated Nude-J mice survived and bred well under the specific pathogen-free conditions. Like nude mice, the Nude-J mice lacked body hair (Figure 1A). To confirm the predicted immunophenotype of Nude-J mice, single-cell suspensions from spleen cells were labeled with fluorescent antibodies against mouse DX-5 (pan NK marker), CD122 (IL-2Rβ), CD3 (T cell marker) and CD19 (B cell marker). Nude mice showed CD3-positive mature T lymphocyte deficiency, whereas B lymphocytes (CD20 positive) and NK cells (DX-5 and CD122 double-positive cells) were detected. In contrast to wild-type mice and nude mice, no T lymphocytes or NK cells were detected in Nude-J mice. BALB/c Nude Rag-2/Jak3 double deficient (Nude RJ) mice showed no T or B lymphocytes or NK cells, as previously reported (Ono et al., 2011) (Figure 1B). Hematoxylin & Eosin staining of the spleen showed that Nude-J mice and Nude mice keep follicular structure in spleen in contrast to marked reduction of follicles in Nude RJ mice (Figure 1C). Thus, Nude-J mice showed a NK cell and T lymphocyte deficient phenotype. 

*Tumor cell engraftment*

**Figure 2:**
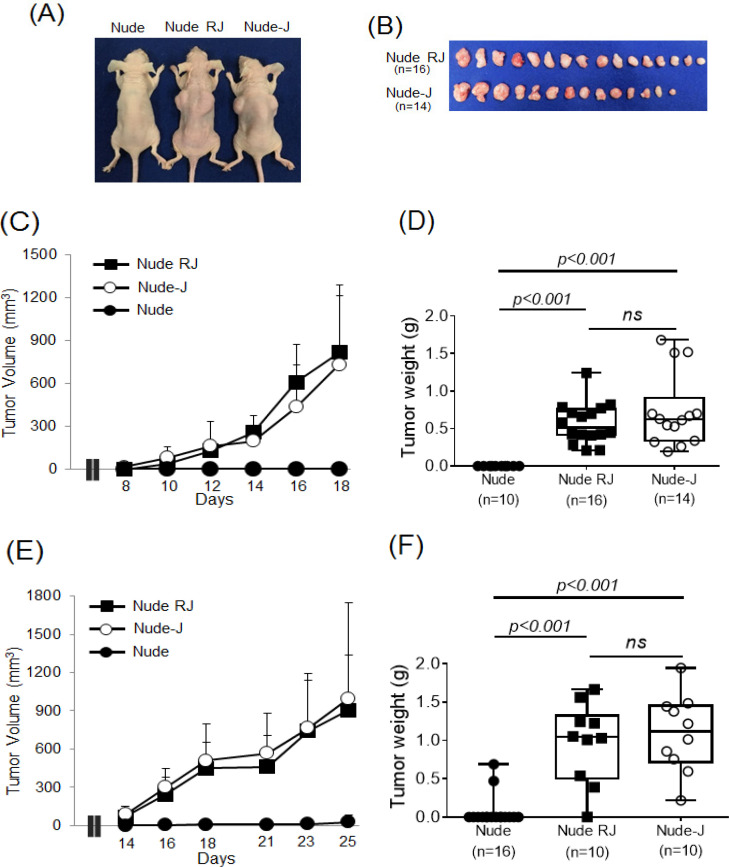
Efficient Development of Tumor cCells in Nude-J Mice. (A) A representative photograph of BCBL-1-bearing mice 18 days after subcutaneous inoculation with 1×107 BCBL-1. (B) Photograph of BCBL-1 tumor tissue. (C) Time course of BCBL-1 tumor volume. (D) BCBL-1 tumor weight of each mouse on day 18. (E) Time course of Z138 tumor volume. (F) Z138 tumor weight of each mouse on day 25

The efficiency of Nude-J mice for engrafting human hematopoietic malignancies was compared with those of Nude RJ and nude mice. Subcutaneous administration of BCBL-1 primary effusion lymphoma cells (1×10^7^ cells) in both flanks resulted in solid tumor formation in both Nude-J (7 mice, 14 sites) and Nude RJ (8 mice, 16 sites) mice, but not in nude (5 mice, 10 sites) mice (Figure 2A-D). Subcutaneous solid tumors were almost the same size in Nude-J mice as in Nude RJ mice (Nude-J vs. Nude RJ: 731.2 ± 554.9 mm3 vs. 816.6 ± 399.5 mm3, p>0.05), whereas no tumor growth was observed in nude mice (Figure 2C). The mice were sacrificed and subcutaneous tumors were removed and weighed on day 18. The weights of BCBL-1 solid tumors in Nude-J mice and Nude RJ mice were also almost the same (Nude-J vs. Nude RJ: 0.73 ± 0.47 g vs. 0.57 ± 0.26 g, p>0.05) (Figure 2B, D). 

Next, the Z138 mantle cell lymphoma cell line (1×10^7^ cells) was subcutaneously administered to these mice. The growths of subcutaneous tumors in Nude-J (5 mice, 10sites) mice and Nude RJ (5 mice, 10 sites) mice were almost the same (Nude-J vs. Nude RJ: 998.5 ± 752.5 mm^3^ vs. 904.9 ± 432.1 mm^3^, p>0.05) (Figure 2E). The mice were sacrificed and subcutaneous tumors were removed and weighed on day 25. The weights of Z138 solid tumors in Nude-J mice and Nude RJ mice were nearly the same (Nude-J: 1.09 ± 0.48 g, Nude RJ: 0.97 ± 0.49 g, p >0.05) (Figure 2F). There was no tumor growth in 7 nude mice while one mouse (2 sites) formed small masses (Figure 2E and 2F). These results indicate that NK cells play critical role for human tumor rejection.

*Direct cytotoxicity by mouse splenocytes*

**Figure 3:**
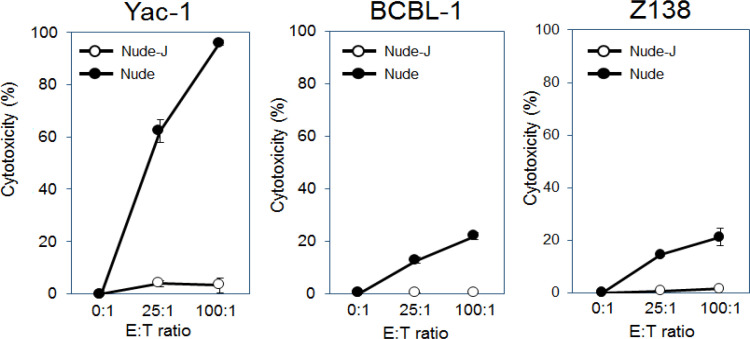
Lack of Cytotoxic Activity of Nude-J Mice Splenocytes. Direct cytotoxic activities of Nude-J and nude mice were compared. Yac-1, BCBL-1 and Z138 Cells were labeled with CFSE and co-cultured with IL-2 stimulated spleen cells for 4 h. Percent cytotoxicity was calculated as [(Dead target cells (%) - spontaneous death (%))/(100 - spontaneous death)] ×100%. The experiments were carried out in triplicate. The values are shown as mean ± SEM. One representative result from 3 independent experiments

Yac-1, BCBL-1 and Z138 Cells were labeled with carboxyfluorescein diacetate succinimidyl ester (CFSE, Dojindo, Kumamoto, Japan) and co-cultured with murine IL-2 stimulated splenocytes. Yac-1 cells (MHC class I negative mouse lymphoma cells) were used as a positive control (Okada et al., 2008), and dead target cells were detected as CFSE+ Ghost Dye 780+ cells by flow cytometry. As shown in Figure 3, Nude-J mice splenocytes showed very low cytotoxic activity against Yac-1, BCBL-1 and Z138 cells, whereas nude mice splenocytes showed high cytotoxic activity. These results indicate that direct cytotoxic activity (NK activity) is absent in Nude-J mice.

**Table 1:** Percentage of Immunocompetent Cells in Nude-J Mice Spleen

Strain	T cells (CD3+)	B cells (CD19+)	NK cells (CD122+DX5+)	Number of mice
BALB/c	24.5 ± 3.4	65.3 ± 4.5	5.2 ± 2.9	5
Nude	1.4 ± 1.4	83.6 ± 6.9	6.7 ± 1.3	5
Nude-J	0.1 ± 0.1	56.7 ± 16.6	0.3 ± 0.2	5
Nude RJ	0.2 ± 0.2	0.3 ± 0.05	0.2 ± 0.2	5

## Discussion

In the present study, we developed and characterized nude mice with a BALB/c background that completely lack NK cells (Nude-J mice) by crossing BALB/c Nude mice and BALB/c Jak3 deficient mice. This lack of NK cells improved the efficiency of xenografts of human malignant cells, indicating that NK cells play an important role in xenografting. 

Nude mice were discovered in 1962 as the first natural immunocompromised mouse (Flanagan, 1966), and have since been used as recipients for human tumor xenografts (Bellet et al., 1979). The nude mouse’s lack of hair allows for easy measurement of subcutaneous tumor size and fluorescent detection of tumors. However, the success rate of human tumor cell transplantation, especially hematological malignancies, has not been satisfactory, since nude mice have high NK activity and leakage of T cells with age (Okada et al., 2019). Attempts to reduce the NK activity in nude mice resulted in the establishment of NIH type 2 nude mice, nude-beige mice and NOD/Scid nude mice. However, the take rate of human tumors was not obviously improved. Recently, genetic modifications have enabled us to establish a complete loss of NK cells in mice, resulting in severely compromised mice such as NOD/Scid common γ-deficient (NOG, NSG mice) (Yahata et al., 2002; Ishikawa et al., 2005) and Jak3-deficient (NOJ) mice (Okada et al., 2008). These mice show improved xenografting of human primary and tumor cells, and are frequently used in human stem cell and tumor cell studies (Shultz et al., 2007). We also established Nude RJ mice having complete loss of B and T lymphocytes and NK cells; these have been used as the recipients of human cell line derived xenografts (CDX) (Kariya et al., 2014). In the present study we established Nude-J mice, and showed that T lymphocytes and NK cell deficiency is essential for the success of human tumor engraftment. Xenotransplantation of human hematological malignancies is relatively difficult compared with solid tumors. Nevertheless, transplantation of human lymphoid malignant cell lines (BCBL-1 and Z138) was successful in Nude-J and Nude RJ mice, but not in Nude mice in our study (Figure 2), indicating that T lymphocytes and NK cells but not B lymphocytes play critical role for xenograft of hematological malignancies. As it is known that Nude mice have equal amount of Ig-M with normal mice (Bloemmen and Eyssen, 1973; Mink et al., 1980), and Nude mice can produce viral specific IgG (Suckling et al., 1982), the presence of B lymphocytes in Nude and Nude-J mice may prevent the mice from some infection in these highly mice. Thus, Nude-J mice have benefit as the recipient of xenotransplantation as relatively resistant to infection compared with Nude RJ mice.

Functional analysis of splenocytes from these mice showed killing activity by Nude mice splenocytes, indicating that mice NK cells can kill human lymphoma cells *in vitro*. On the other hand, no cytotoxic activity against human lymphoma cell (BCBL-1 and Z138) was shown in Nude- J mice (Figure 3). Unexpectedly, Nude-J mice showed weak cytotoxic activity against Yac-1 (mouse lymphoma cell lines) cells. We speculate that it may due to phagocytosis by macrophages. Since Yac-1 is derived from a new born A/Sn mouse and Nude mice were in BALB/c background, BALB/c mice macrophage may react with Yac-1 cells.

Patient-derived xenograft (PDX) models are created by the engraftment of patient tumor tissues into immunocompetent mice, and have become a standard “Avatar” model of human cancer (Tentler et al., 2012); (Okada et al., 2018). Nude mice have been used as recipients of PDX, but the transplantation efficiency was not satisfactory (Kelland, 2004). NOD/Scid based immunocompromised mice (NOG, NSG) mice are now frequently used as the recipients of PDX, since these mice show combined immunodeficiency and high xenograft efficiency compared with nude mice (Okada et al., 2019). However, since Scid mice are based on a mutation of DNA-dependent protein kinase, catalytic subunit (DNA-PKcs) (Peterson et al., 1995; Jeggo et al., 1996), NOD/Scid mice have a DNA damage repair deficiency of the somatic cells as well as T and B lymphocytes. Thus, NOD/Scid based immunocompromised mice are not an appropriate recipient of PDX for the evaluation of drug sensitivity, because they are very sensitive to DNA damage factors such as irradiation and chemotherapeutic drugs. In contrast, as Nude-J mice have normal DNA-PKcs activity, they show resistance to DNA damage comparable to that of humans. In addition, their lack of hair allows easy detection of subcutaneous tumors. Nude-J mice are therefore the ideal recipients for anti-cancer drug screening using PDX and cell line derived xenografts (CDX).

In conclusion, we established nude mice with complete loss of NK cells, BALB/c Jak3 deficient (Nude-J) mice, and showed that NK cells play a crucial role in the rejection of human tumor cells. Nude-J mice are optimal for human tumor engraftment, especially hematological malignancies, and can be a useful tool for human cancer research. 

## Author Contribution Statement

The authors confirm contribution to the paper as follows: study conception and design: Jutatip Panaampon, Kenichi Sasamoto, Seiji Okada; data collection: Jutatip Panaampon, Kenichi Sasamoto; analysis and interpretation of results: Jutatip Panaampon, Kenichi Sasamoto, Ryusho Kariya; draft manuscript preparation: Jutatip Panaampon, Kenichi Sasamoto: Review & editing of manuscript: Seiji Okada. All authors reviewed the results and approved the final version of the manuscript.

## References

[B1] Bellet RE, Danna V, Mastrangelo MJ (1979). Evaluation of a “nude” mouse-human tumor panel as a predictive secondary screen for cancer chemotherapeutic agents. J Natl Cancer Inst.

[B2] Bloemmen J, Eyssen H (1973). Immunoglobulin levels of sera of genetically thymusless (nude) mice. Eur J Immunol.

[B3] Flanagan SP (1966). ‘Nude’, a new hairless gene with pleiotropic effects in the mouse. Genet Res.

[B4] Ishikawa F, Yasukawa M, Lyons B (2005). Development of functional human blood and immune systems in NOD/SCID/IL2 receptor {gamma} chain(null) mice. Blood.

[B5] Jeggo PA, Jackson SP, Taccioli GE (1996). Identification of the catalytic subunit of DNA dependent protein kinase as the product of the mouse scid gene. Curr Top Microbiol Immunol.

[B6] Kariya R, Matsuda K, Gotoh K (2014). Establishment of nude mice with complete loss of lymphocytes and NK cells and application for in vivo bio-imaging. In Vivo.

[B7] Kelland LR (2004). Of mice and men: values and liabilities of the athymic nude mouse model in anticancer drug development. Eur J Cancer.

[B8] Mink JG, Radl J, van den Berg P (1980). Serum immunoglobulins in nude mice and their heterozygous littermates during ageing. Immunology.

[B9] Nehls M, Pfeifer D, Schorpp M (1994). New member of the winged-helix protein family disrupted in mouse and rat nude mutations. Nature.

[B10] Okada S, Harada H, Ito T (2008). Early development of human hematopoietic and acquired immune systems in new born NOD/Scid/Jak3(null) mice intrahepatic engrafted with cord blood-derived CD34 (+) cells. Int J Hematol.

[B11] Okada S, Vaeteewoottacharn K, Kariya R (2018). Establishment of a patient-derived tumor xenograft model and application for precision cancer medicine. Chem Pharm Bull.

[B12] Okada S, Vaeteewoottacharn K, Kariya R (2019). Application of highly immunocompromised Mice for the establishment of Patient-Derived Xenograft (PDX) Models. Cells.

[B13] Ono A, Hattori S, Kariya R (2011). Comparative study of human hematopoietic cell engraftment into BALB/c and C57BL/6 strain of rag-2/jak3 double-deficient mice. J Biomed Biotechnol.

[B14] Park SY, Saijo K, Takahashi T (1995). Developmental defects of lymphoid cells in Jak3 kinase-deficient mice. Immunity.

[B15] Peterson SR, Kurimasa A, Oshimura M (1995). Loss of the catalytic subunit of the DNA-dependent protein kinase in DNA double-strand-break-repair mutant mammalian cells. Proc Natl Acad Sci U S A.

[B16] Shultz LD, Goodwin N, Ishikawa F (2014). Human cancer growth and therapy in immunodeficient mouse models. Cold Spring Harb Protoc.

[B17] Shultz LD, Ishikawa F, Greiner DL (2007). Humanized mice in translational biomedical research. Nat Rev Immunol.

[B18] Suckling AJ, Jagelman S, Webb HE (1982). Immunoglobulin synthesis in nude (nu/nu), nu/+ and reconstituted nu/nu mice infected with a demyelinating strain of Semliki Forest virus. Clin Exp Immunol.

[B19] Suzuki K, Nakajima H, Saito Y (2000). Janus kinase 3 (Jak3) is essential for common cytokine receptor gamma chain (gamma(c))-dependent signaling: comparative analysis of gamma(c), Jak3, and gamma(c) and Jak3 double-deficient mice. Int Immunol.

[B20] Tentler JJ, Tan AC, Weekes CD (2012). Patient-derived tumour xenografts as models for oncology drug development. Nat Rev Clin Oncol.

[B21] Vainchenker W, Dusa A, Constantinescu SN (2008). JAKs in pathology: role of Janus kinases in hematopoietic malignancies and immunodeficiencies. Semin Cell Dev Biol.

[B22] Yahata T, Ando K, Nakamura Y (2002). Functional human T lymphocyte development from cord blood CD34+ cells in nonobese diabetic/Shi-scid, IL-2 receptor gamma null mice. J Immunol.

